# Skin-associated adipocytes in skin barrier immunity: A mini-review

**DOI:** 10.3389/fimmu.2023.1116548

**Published:** 2023-01-24

**Authors:** Jingyan Guan, Congxiao Wu, Yunfan He, Feng Lu

**Affiliations:** Department of Plastic and Cosmetic Surgery, Nanfang Hospital, Southern Medical University, Guangzhou, Guangdong, China

**Keywords:** skin barrier immunity, adipocytes, adipokines, antimicrobial peptides, infection

## Abstract

The skin contributes critically to health *via* its role as a barrier tissue against a multitude of external pathogens. The barrier function of the skin largely depends on the uppermost epidermal layer which is reinforced by skin barrier immunity. The integrity and effectiveness of skin barrier immunity strongly depends on the close interplay and communication between immune cells and the skin environment. Skin-associated adipocytes have been recognized to play a significant role in modulating skin immune responses and infection by secreting cytokines, adipokines, and antimicrobial peptides. This review summarizes the recent understanding of the interactions between skin-associated adipocytes and other skin cells in maintaining the integrity and effectiveness of skin barrier immunity.

## 1 Introduction

As the largest organ and outermost layer of the human body, the skin serves as the first-line defense against various external pathogenic factors, including physical, chemical, and biological stresses, and plays a pivotal role in preventing dehydration. Maintenance of these functions relies primarily on a sound skin barrier; any functional or structural defect of the skin barrier may induce various skin diseases such as atopic dermatitis (AD) (1) and psoriasis (2). In addition to the skin physical barrier, which mainly consists of keratinocytes and their products, skin barrier immunity is also recently found to play an important role in maintaining integrity of skin barrier (3). Recent studies found that skin-resident immune cells, including Langerhans cells (LCs), dendritic cells (DCs), innate lymphoid cells (ILCs), and T cells, work along with the skin-resident structural cells, such as keratinocytes and fibroblasts to protect the homeostatic balance of skin barrier immunity (4). The integrity of skin barrier closely depends on the homeostasis of skin barrier immunity and challenged when homeostasis is irreversibly compromised. During the last few years, it has been recognized that skin-associated adipocytes, which located in the subcutis and at the bottom of the dermis, may play important roles in modulating skin immunity by producing various cytokines, adipokines, and antimicrobial peptides (AMPs) (5). Studying of the effect of skin-associated adipocytes on skin barrier immunity may deepen our understanding of skin barrier system (4, 6, 7). Therefore, this mini-review will summarize the recent developments and current understanding of how the skin-associated adipocytes mediate multiple facets of skin barrier immunity.

## 2 Composition of skin barrier and location of skin-associated adipocytes

Skin barrier can be functionally divided into four levels, namely, the microbiome barrier, the chemical barrier, the physical barrier, and the immune barrier (8). As the outermost layer, the microbiome barrier is composed of abundant microbial communities which act as the first line of defense. The commensal microbes play important roles in inhibiting the colonization of pathogenic bacteria (9) and in suppressing inflammatory cytokines released by skin-resident cells (10). The chemical barrier commonly refers to a series of protective molecules produced by different types of skin cells, including AMPs (11), natural moisturizing factors (NMFs) (12), epidermal lipids (13), and cutaneous pH-contributing factors. With regard to the physical barrier, the stratum corneum (SC) and tight junctions (TJs) are the most important components. The SC consists of several layers of denucleated and flattened cornified cells, which result from the terminal differentiation of keratinocytes (14), the TJs are transmembrane proteins expressed by keratinocytes in the stratum granulosum (SG) and function as connections between adjacent keratinocytes (15). The immune barrier is composed of various resident immune cells in the epidermis and dermis. In addition, other skin structural cells exert immunologic functions and work together with immune cells to maintain immune barrier integrity. These four elements of the skin barrier are not independent of one another. Instead, they are inextricably interconnected, and each element influences the others. Any dysfunction in the barrier compartment may lead to a vicious circle, compounding damage to the barrier and resulting in various dermatoses.

The skin consists of several distinct layers and a series of cutaneous appendages, of which the epidermis forms the outermost layer and accounts for the majority of the barrier function. The next layer is the dermis, which can be subdivided into papillary and reticular dermis (16). Skin-associated adipocytes reside in the reticular dermis and subcutis, where they form dermal white adipose tissue (dWAT) and subcutaneous white adipose tissue (sWAT), respectively. In mice and rats, these two layers are clearly separated by a thin layer of skeletal muscle called the *panniculus carnosus* (17). However, in human skin, there is no distinct demarcation between dWAT and sWAT, and the existence of dWAT has not yet been confirmed because of the difficulty in tracing (18).

## 3 The immunoregulating potential of skin-associated adipocytes

WAT used to be merely considered as an energy storage site with simple functions, such as mechanical protection and thermal insulation (6). However, recent studies demonstrated that adipose tissue acts as an endocrine organ with a pivotal role in the regulation of immune responses (19). Adipocyte is the most common cell type in WAT; however, adipose tissue also contains a variety of immune cells. The cross-talk between adipocytes and immune cells remarkably affects the local inflammatory state. For instance, receptors for the macrophage-derived factors interleukin (IL)-1β and tumor necrosis factor (TNF), as well as receptors for the cytokine IL-17 produced by helper T (Th) 17 cells, are expressed on the surface of adipocytes and mediate downstream pro-inflammatory signals (20, 21). In addition, receptors for the anti-inflammatory cytokine IL-10 are enriched in mature adipocytes and contribute to the creation of an anti-inflammatory milieu (22). Moreover, adipocytes produce an array of inflammatory cytokines and chemokines, including several ILs, notably IL-6, IL-1β, TNF-α, and monocyte chemoattractant protein-1 (MCP-1) (23, 24). In addition, the immunoregulatory potency of skin-associated adipocytes depends on adipokines, a series of small bioactive proteins produced by adipocytes (25). Based on their inflammatory properties, adipokines can be classified as pro-inflammatory and anti-inflammatory (26, 27) (**Table 1**).

**Table 1:** Adipokines in the skin and their effects.

Adipokine	Properties	Effects	Ref.
Leptin	Pro-inflammatory	Promotes pro-inflammatory biomolecules productionin skin constructive cells	(28–31)
		Changes secretion profile of immune cells	(32–34)
	Antibacterial	Assists removal of Streptococcus pneumoniae	(35)
		Promotes hBD-2 production of human keratinocytes	(36)
Chemerin	Pro-inflammatory	Acts as a chemoattractant	(37–39)
		Facilitates pro-inflammatory cytokines production	(40–42)
	Anti-inflammatory (need further confirm)	Suppresses neutrophil and monocyte recruitment	(43)
	Antibacterial	Possesses a certain degree of bactericidal capacity on E. coli and K. pneumoniae	(44)
Visfatin	Pro-inflammatory	Activates human leukocytes and induces cytokine production	(45, 46)
		Stimulates chemokines production	(47)
	Antibacterial	Promotes AMPs production of human keratinocytes	(48)
ZAG	Pro-inflammatory	Modulates immune responses	(49)
	Others	Promotes keratinocytes terminal differentiation	(49)
Resistin	Pro-inflammatory	Promotes pro-inflammatory cytokines production in other diseases	(27, 50)
		Not fully been elucidated in skin diseases	
Adiponectin	Anti-inflammatory	Suppresses TNF and IFN-γ production	(51, 52)
		Promotes anti-inflammatory cytokines production	(53)
		Suppresses TNF and IFN-γ production	(53)
	Others	Promotes epidermal cells proliferation and migration	(54)
		Enhances skin lipid synthesis	(55)
		Enhances filaggrin expression	(56)
		Impedes UV-induced dermal matrix degradation	(57)
		Anti-fibrosis	(58)
CTRP-3	Anti-inflammatory	Suppresses pro-inflammatory pathways in monocytes and adipocytes	(59, 60)
		Suppresses chemokines secretion	(60)

### 3.1 Pro-inflammatory adipokines

Leptin, one of the most abundant pro-inflammatory adipokines expressed by skin adipocytes (61), and the first to be discovered, has been extensively investigated in skin biology (62). The Janus kinase/signal transducer and activator of transcription (JAK/STAT) signaling pathway is the main signaling pathway activated by leptin (63). Moreover, other inflammatory signaling pathways, including the mitogen-activated protein kinase (MAPK), phosphoinositide 3-kinase (PI3K), PPAR gamma coactivator/peroxisome proliferator-activated receptor (PGC/PPAR), adenosine monophosphate kinase (AMPK), and extracellular signaling-regulated kinase 1/2 (ERK1/2) pathways can be stimulated by leptin (64). In addition to adipocytes, leptin is secreted by keratinocytes, fibroblasts, and sebocytes. These cells also possess leptin receptors and, therefore, respond to leptin in addition to secreting it (65–67). *In vitro* studies have demonstrated that leptin treatment can induce or enhance the production of pro-inflammatory cytokines (such as TNF-α, IL-6, and IL-8) and the expression of inflammatory enzymes (cyclooxygenase [COX]-2 and 5-lipooxygenase [LOX]) in human keratinocytes, synovial fibroblasts, and SZ95 sebocytes (28–31). Moreover, leptin affects the secretion profile of multiple immune cells, such as monocytes/macrophages, neutrophils, natural killer (NK) cells, eosinophils, basophils, and T cells, which would further aggravate pro-inflammatory processes (32–34).

Chemerin, for which receptors are widely present on the surface of immune cells including T cells, NK cells, DCs, monocytes/macrophages, and neutrophils, is an adipokine that is considered as chemoattractant and modulator of the immune response (37). Chemerin has a stronger chemotactic potency in NK cells and DCs migration than the classic chemokines C-X-C motif chemokine ligand (CXCL) 8 and CXCL12 (approximately 20-fold and 100-fold stronger, respectively) (38, 39). In the human skin, chemerin is expressed in both the epidermis and dermis, but its distribution varies between healthy and diseased skin. In healthy skin, chemerin is mainly produced by keratinocytes and rarely occurs in the dermis. In contrast, the skin of patients with psoriasis shows decreased chemerin expression in keratinocytes and an abnormal increase in chemerin expression in the dermis (68). Although many reports have confirmed the pro-inflammatory characteristics of chemerin in facilitating the secretion of pro-inflammatory cytokines such as TNF-α, IL-1β and IL-6 (40–42), Cash et al. reported its anti-inflammatory effects (43). In a mouse model of zymosan-induced inflammation, neutrophils and monocytes were suppressed by chemerin in a chemokine-like receptor 1-dependent manner. However, since another study ruled out the direct anti-inflammatory effect of chemerin on macrophages ex vivo (69), thereby emphasizing that more investigations are needed to confirm the anti-inflammatory capacity of chemerin.

Visfatin, also known as pre-B-cell colony-enhancing factor (PBEF), has been identified as an adipokine in the last two decades (70). As a pro-inflammatory adipokine, visfatin upregulates the secretion of pro-inflammatory cytokines (such as IL-6, TNF-α, and IL-1β) in human monocytes and endothelial cells (45), as well as increasing the expression of co-stimulatory molecules (CD80, CD40, and intercellular cell adhesion molecule-1 (ICAM-1)] in monocytes to enhance the activation of T cells *via* the p38 and mitogen-activated/extracellular response kinase kinase (MEK)-mediated pathways (46). In the skin, visfatin stimulates the production of a series of chemokines, including CXCL8, CXCL10, and C-C motif chemokine ligand (CCL) 20, *via* the nuclear factor-kappa-gene binding (NF-κB) and STAT3 pathways in keratinocytes under the synergistic effect of TNF-α (47).

Upregulation of pro-inflammatory adipokines in the skin usually causes an abnormal inflammatory state and disordered cutaneous metabolism. However, some adipokines with pro-inflammatory properties have been demonstrated to play important roles in skin barrier homeostasis, and their expression usually decreases in patients with skin diseases. Zinc alpha (2)-glycoprotein (ZAG) is considered an important factor for keratinocyte terminal differentiation and immune response modulation, and its expression in the skin of patients with AD and psoriasis is significantly lower than that in healthy controls (49, 71). Moreover, topical treatment with ZAG resulted in the relief of immune abnormalities and recovery of skin barrier function (49), which further indicated the role of ZAG in skin barrier immunity. Another pro-inflammatory adipokine, resistin was found to promote the expression of many pro-inflammatory cytokines (such as TNF-α and IL-6) (27, 50) and adhesion molecules [such as ICAM-1 and vascular cell adhesion molecule 1 (VCAM-1)] (27) in many diseases, such as vascular diseases (72), metabolic disorders (73), and cystic fibrosis (74). However, an investigation focused on the association between resistin and AD indicated that the serum resistin levels in patients with AD were significantly lower than those in healthy subjects (75); the underlying mechanisms have not yet been elucidated.

### 3.2 Anti-inflammatory adipokines

Although present in smaller amounts than pro-inflammatory adipokines, certain anti-inflammatory adipokines are also found in the skin. Their role in skin barrier immunity is being intensively investigated.

Adiponectin, which interacts with the cellular receptors ADIPOR1 and ADIPOR2, is the most extensively studied anti-inflammatory adipokine. Through cellular receptors, adiponectin activates AMPK, peroxisome PPARα, and p38 MAPK (76). Adiponectin is also secreted by keratinocytes and sebocytes in the skin (77). The anti-inflammatory properties of adiponectin are reflected in the following aspects. First, adiponectin is capable of inhibiting TNF production by suppressing the NF-κB kinase signaling (51, 52). Second, adiponectin stimulates the production of anti-inflammatory cytokines (such as IL-10 and IL-1 receptor antagonist [IL-1RA]) by human macrophages, monocytes, and DCs, and suppresses interferon-gamma (IFN-γ) secretion by lipopolysaccharide (LPS)-stimulated human macrophages (53). In addition, the inhibitory effect of adiponectin on T-cell response and macrophage phagocytosis was demonstrated in the same study (53). In skin-resident cells, adiponectin receptors are present on the surface of keratinocytes, fibroblasts, sebocytes, and melanocytes (78, 79). In addition to immune modulation, adiponectin is responsible for other functions. *In vitro* and *in vivo* experiments have indicated that adiponectin promotes the proliferation and migration of epidermal cells *via* the ERK signaling pathway, thereby facilitating skin wound healing (54). Additionally, adiponectin enhances skin lipid synthesis and filaggrin expression *via* silent information tegulator 1 (SIRT1) signaling (55, 56), thereby aiding the maintenance of skin barrier homeostasis. Moreover, studies focusing on the dermis have indicated that adiponectin may possess anti-fibrotic potency and resistance to UV-induced dermal matrix degradation (57, 58).

C1q/TNF-related Protein-3 (CTRP-3) is an adipokine with a structure homologous to adiponectin (80). CTRP-3 possesses strong potency in suppressing common pro-inflammatory pathways in monocytes and adipocytes, such as toll like receptor (TLR), LPS, and fatty acid-mediated inflammation (59, 60). CTRP-3 is, therefore, classified as an anti-inflammatory adipokine. In primary monocytes of healthy humans, CTRP-3 was found to suppress the LPS-stimulated secretion of macrophage migration inhibitory factor (MIF), CCL4, MCP-1, and the lauric acid-stimulated release of TNF and IL-6 (60). In adipocytes, CTRP-3 inhibits the secretion of MCP-1, which is normally induced by various TLR agonists (60).

## 4 Skin-associated adipocytes maintain skin barrier immunity by exerting antimicrobial capacity

Skin-derived AMPs are small peptides secreted primarily by keratinocytes that form the first line of defense against microbial pathogens (11). They act as multifunctional biomolecules that maintain the integrity and stability of the skin barrier in several ways. The AMPs exert antibiotic-like activity that directly kills pathogens (81) and act as effector molecules that play a vital role in balancing immune responses and modulating cell activities (82, 83). Recent studies have identified the role of skin-associated adipocytes in barrier immunity as a significant source of AMPs. When the skin suffers from inflammation, injury, and infection, the lesion site enters a state called reactive adipogenesis, characterized by a local increase in dermal adipocytes from which AMPs are responsively produced to suppress infection (84, 85). Using a *Staphylococcus aureus*-induced skin infection mouse model, Zhang et al. (86) indicated that newly differentiating preadipocytes could directly contribute to host defense by producing cathelicidin, which is one of the best characterized AMPs. This defense effect was inhibited in Zfp423-deficient mice and in the mice treated with inhibitors of PPAR-γ, which further confirmed the antimicrobial role of adipocytes. The mechanisms by which adipocytes recognize and respond to bacteria were subsequently uncovered possibly involving a TLR2-mediated pathway (87).

In addition to classic AMPs, some adipokines secreted by skin-associated adipocytes exert antimicrobial activities. In a study by Mancuso et al. (35), the authors found that low levels of circulatory leptin induced by starvation resulted in the impairment of bacterial clearance in a *Streptococcus pneumoniae* infected mouse model. This defective function was restored by administration of exogenous leptin, indicating the antimicrobial ability of leptin. Structural homology between chemerin and cathelicidin has been predicted (40, 88). Based on this prediction, Kulig et al. (44) tested the antibacterial capacity of chemerin against *Escherichia coli* and *Klebsiella pneumoniae*. The results showed that although the antimicrobial effects of chemerin on *E. coli* and *K. pneumoniae* were less potent than those of the classic AMP LL-37, chemerin displayed a certain degree of bactericidal capacity, especially the isoforms truncated by the cysteine proteases cathepsin L and K.

Moreover, skin-associated adipocytes can induce AMP secretion from other skin cells *via* adipokines. Leptin can enhance the production of human beta-defensin-2 (hBD-2) in human keratinocytes *in vitro* (36). Another *in vitro* experiment showed that visfatin activated the secretion of a series of AMPs, including cathelicidin, S100A7, hBD-2, and hBD-3, in normal human keratinocytes (48).

## 5 Potential role of adipocyte-derived lipids in maintaining skin barrier homeostasis

Skin surface lipids are comprised of extracellular and sebaceous lipids. Extracellular lipids, also called keratinocyte-derived lipids, are found predominantly in the SC, and mainly consist of ceramides (45–50%), cholesterol (25%), and free fatty acid (10–15%) (13). Lipid precursors and lipid synthases are secreted by lamellar bodies, from which extracellular lipids are synthesized in the extracellular spaces of the stratum corneum. Sebaceous lipids are sebum molecules secreted by sebaceous glands and include cholesteryl esters, triglycerides, squalene, and wax esters. Together with sweat secreted by sweat glands, sebaceous lipids form a hydrolipidic film on the skin surface that plays a role in skin protection. Lipids are important components of the physical barrier of the skin. Additionally, some skin lipids contribute to skin barrier maintenance by acting as bioactive mediators in cutaneous immunity and inflammation (89). Alteration of skin lipid contents and compositions can cause skin barrier dysfunction and skin immunity dysregulation, resulting in various dermatoses such as psoriasis (90), AD (91), and acne vulgaris (92).

Adipocytes, being the energy storage vault of the body, store excess energy in the form of triglycerides. In addition, adipocytes contain many ceramides and a number of other lipid species. Numerous studies have investigated the role of adipocyte-derived lipids in the development of multiple metabolic diseases such as diabetes, insulin resistance, cardiomyopathy, atherosclerosis, and hepatic-steatosis (93). Whereas the effect of adipocyte-derived lipids on the skin barrier has seldom been studied. Although most of the studies that focused on metabolic diseases have indicated the deleterious effects resulting from excessive accumulation of triglycerides and ceramides in adipocytes. In view of the particular role of lipids in the maintenance of the skin barrier, adipocyte-derived lipids might, to some extent, possess the capacity for skin barrier protection.

## 6 Conclusions and future perspectives

**Figure 1:**
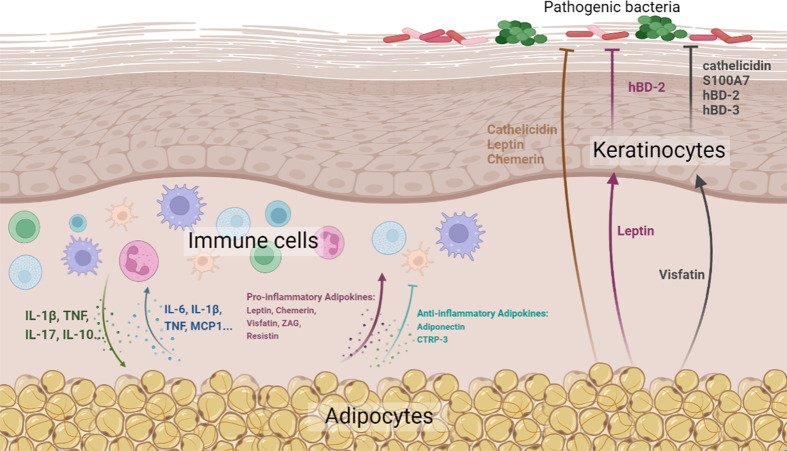
Diagrammatic representation of the effects of skin-associated adipocytes on skin barrier immunity (Created with BioRender.com. Agreement NO. QN24VPBCG8).

In this review, we attempted to summarize and highlight the important role of skin-associated adipocytes in skin barrier immunity, either by the production of biomolecules (cytokines, adipokines, and AMPs) or by direct interaction with skin-resident cells (**Figure 1**). In addition, the abundant storage of lipid species in skin-associated adipocytes may indicate a potential role in skin barrier maintenance. The pathogenesis of inflammatory skin diseases, such as AD and psoriasis, involves complex interactions between abnormal immunity, dysbacteriosis, and skin barrier defects, indicating therapeutic agents with comprehensive efficacy are required. On account of the comprehensive effects of skin-associated adipocytes on skin barrier immunity, they might be potential therapeutic agents for this kind of skin disease. As a matter of fact, the therapeutic effect of preadipocytes (adipose-derived mesenchymal stem cells) has been determined by various research teams. However, the effects of adipocytes on immunity are two-sided. Although an increasing number of anti-inflammatory biomolecules have been identified in adipocytes, adipocyte-derived pro-inflammatory factors cannot be ignored. Therefore, pretreatment of adipose tissue or adipocytes, such as by extracting anti-inflammatory components and excluding pro-inflammatory elements, would be needed to enhance their effect in maintaining skin barrier homeostasis.

## Author contributions

JG and CW drafted the review. YH and FL revised the manuscript. All authors contributed to the article and approved the submitted version.
